# Experimental Study on Mechanical Properties of Recycled Aggregate Mixed Soil

**DOI:** 10.3390/ma17184448

**Published:** 2024-09-10

**Authors:** Xuliang Wang, Qinxi Dong, Jun Hu, Peng Liu, Zetian Li, Yongzhan Chen, Guoyang Xu

**Affiliations:** 1 School of Civil Engineering and Architecture, Hainan University, Haikou 570228, China; wangxul@hainanu.edu.cn (X.W.); dongqinxi@hainanu.edu.cn (Q.D.); hj7140477@hainanu.edu.cn (J.H.); 22220856000077@hainanu.edu.cn (P.L.); lizetian@hainanu.edu.cn (Z.L.); 2 Collaborative Innovation Center of Marine Science and Technology, Hainan University, Haikou 570228, China; 3 Hainan Posent Geotechnical Engineering Co., Ltd., Haikou 570311, China; 13134437182@163.com

**Keywords:** recycled aggregates, soft soil stabilizers, soil mixes, UCS, FESEM, PSD

## Abstract

In the context of efforts aimed at reducing carbon emissions, the utilization of recycled aggregate soil mixes for soil stabilization has garnered considerable interest. This study examines the mechanical properties of mixed soil samples, varying by dosage of a soft soil curing agent C, recycled aggregate R content, and curing duration. Mechanical evaluations were conducted using unconfined compressive strength tests (UCS), field emission scanning electron microscopy (FESEM), and laser diffraction particle size meter tests (PSD). The results indicate that the strength of the mixed soil samples first increases and then decreases with higher dosages of recycled aggregate, reaching optimal strength at a 20% dosage. Similarly, an increase in curing agent dosage enhances the strength, peaking at 20%. The maximum strength of the mixed soils is achieved at 28 days under various proportions. The introduction of the curing agent leads to the formation of a flocculent structure, as observed in FESEM, which contributes to the enhanced strength of the soil mixes. Specimens prepared with a combination of 20% R and 20% C, maintained at a constant moisture content of 20%, and cured for 28 days exhibit a balance between economic, environmental, and engineering performance.

## 1. Introduction

With the large-scale development of new urban areas and the renovation of existing ones, the generation of construction waste from these projects has emerged as a significant issue. Annually, China produces approximately 2.3 billion tons of construction waste, which constitutes about 40% of the country’s total solid waste output. This substantial volume of construction waste not only poses environmental challenges but also conflicts with the principles of green and sustainable development, a concern that is increasingly recognized worldwide [1,2,3].

Currently, various countries and regions have implemented differing methods and policies for managing construction waste, all aimed at enhancing the efficiency of its utilization [4,5,6,7]. One aspect of research on construction waste indicates that the utilization of construction waste exerts a certain impact on the surrounding environment [8,9]. However, through recycling, the reuse of recycled aggregates constitutes non-hazardous construction waste. Scholars across different countries have extensively studied and endorsed the benefits of recycled aggregates in terms of carbon emissions and sustainable utilization [10,11,12]. After that, a considerable body of research has been conducted by scholars on the application of recycled aggregates in construction waste. For instance, studies involving the alkali activation of recycled aggregate concrete (AARAC) have demonstrated that the use of recycled fine aggregate (RFA) in AARAC offers acceptable mechanical and durability properties [13]. Additionally, the migration behavior of water within recycled aggregates has been investigated. Researchers have employed two low-field nuclear magnetic resonance (LF-NMR) pulse sequences to study the effects of the water–cement ratio, strength, and water content on recycled aggregates. It has been concluded that in high-strength recycled aggregate concretes, an increase in added water raises the effective water–cement ratio, thereby reducing the compressive strength of the concrete [14]. Due to the unique properties of recycled aggregates, their application has been extensively adopted in the domains of recycled concrete, roads, and foundation backfill, among others. The recycling rate of concrete wastes has been enhanced by some researchers who have employed a method that involves the production of zero-cement hollow blocks through the carbonation and curing of recycled concrete aggregate (RCA) [15]. Additionally, it has been demonstrated by others that the utilization of RCA in the surface layer of road pavements not only increases the recycling rate of concrete wastes but also reduces the reliance on natural aggregates in such projects [16]. Furthermore, the mechanical behavior of steel pipe recycled aggregate concrete (RACFST) columns under fire conditions has been studied by Liu Wenchao and colleagues, providing valuable insights for practical engineering applications [17]. A significant number of scholars have engaged in comprehensive studies, both theoretical and practical, focusing on the properties of recycled aggregate and their applications in actual engineering projects, resulting in notable advancements in the field [18,19,20,21,22,23].

Soft clay, commonly encountered in engineering due to its high plasticity and compressibility instability [24], is typically treated after engineering operations using soil stabilization techniques. One commonly used method is the addition of curing agents, which are favored for their convenient application, low cost, and effective treatment outcomes [25,26,27,28]. In engineering practices, curing agents such as cement and lime are frequently employed to enhance the mechanical properties and increase the foundation’s bearing capacity [29,30,31,32,33]. However, their use is significantly curtailed by drawbacks such as high energy consumption and substantial pollution emissions [34,35,36,37,38,39]. Despite the widespread practical use of recycled aggregates as sustainable materials, research on their mechanical properties when used as synergistic curing agents in clay is relatively sparse. Therefore, this study explores the use of recycled aggregate and soft soil curing agents in combination with red clay to create a hybrid soil, aiming to enhance soil stabilization effectively.

In this paper, the mechanical properties and microscopic mechanisms of soil mixes, composed of recycled fine aggregate, curing agents, and red clay at various dosages and maintenance ages, are investigated. The reinforcing effects of the soil mixes, under the synergistic influence of recycled fine aggregate and curing agents, are analyzed. Microscopic testing methods, including Scanning Electron Microscope (SEM) tests and laser particle size analysis, are employed to elucidate the reinforcing mechanisms of the soil mixes. This study aims to reveal the complex interactions and reinforcement mechanisms within the mixed soil, facilitated by the synergistic effects of the recycled aggregate and curing agent.

## 2. Materials and Methods

### 2.1. Materials

**Figure 1:**
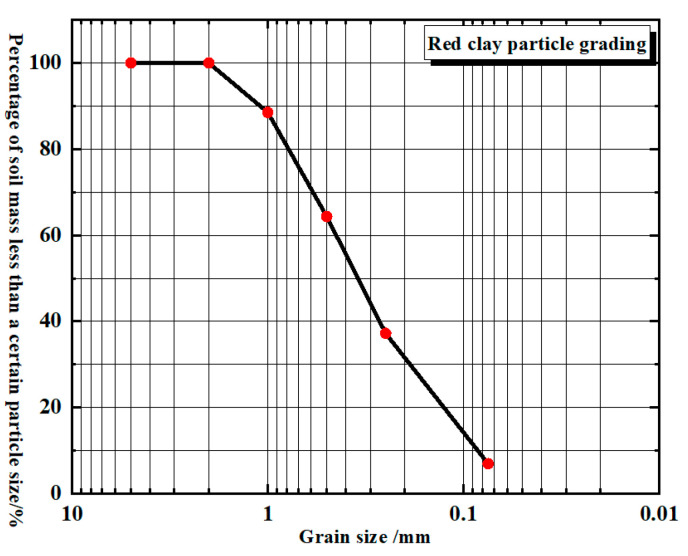
Gradation of red clay particles and the red clay used.

**Figure 2:**
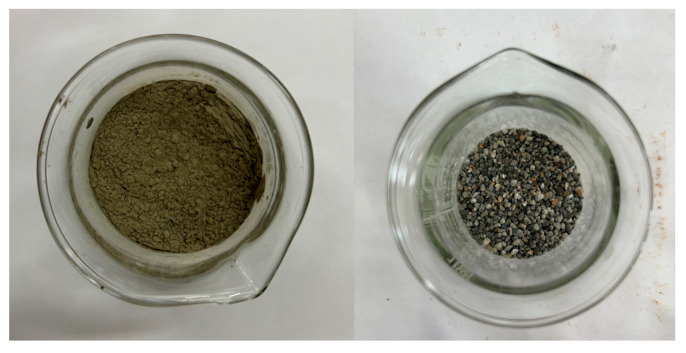
Soft soil stabilizers and recycled aggregate.

**Figure 3:**
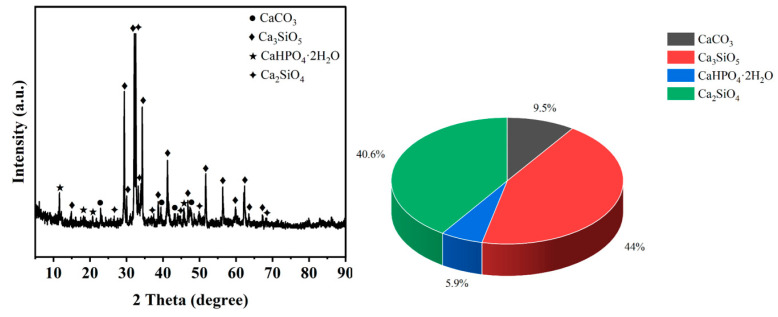
XRD plots of soft soil stabilizers and their compositional percentage.

(a). The test soil utilized in this study originates from a region in Hainan Province and consists of red clay. The soil samples are all remolded. The original soil was dried and crushed, then sieved through a 2 mm mesh. Particles smaller than 2 mm were selected for the study, and their particle size distribution is presented in Figure 1. The properties of the red clay were measured according to the Standard for Geotechnical Test Methods (GB/T 50123-2019), with the results summarized in Table 1. Additionally, the mineral composition of the red clay was analyzed using X-ray diffraction (XRD), revealing that the red clay is predominantly composed of quartz and kaolinite, which account for 61.9% and 38.1% of the chemical composition, respectively.
(b). The recycled aggregates used in the test were sourced from demolished old houses and laboratory waste concrete test blocks. After the manual removal of debris, the materials were processed, crushed, and sieved through a 2 mm screen. Aggregates with a particle size range of 1 mm to 2 mm were selected as one of the raw materials for this test. The recycled fine aggregate and curing agent utilized in the study are depicted in Figure 2.
(c). The curing agent used in the test was a milky-white, powdery soft soil curing agent provided by a company. The chemical composition of the curing agent material was analyzed using XRD, and the results are presented in tables and graphs, as shown in Table 2 and Figure 3.

### 2.2. Sample Preparation

In accordance with the Standard for Geotechnical Test Methods (GB/T 50123-2019), the red clay and recycled aggregate were oven-dried at a constant temperature for 24 h to ensure complete dryness. Following the test program’s ratio, the required amounts of curing agent, red clay, recycled aggregate, and water were weighed and mixed thoroughly by stirring. The mixture was then uniformly poured into a petroleum jelly-coated compaction cylinder in three layers, employing a compaction method. The number of compaction strokes (23) was calculated by controlling the standard compaction energy and the fixed number of layers (3). After removing the three-valve film, the samples were placed in a standard curing room and cured for the duration specified by the test program. This process resulted in the formation of complete cylindrical specimens with a diameter of 39.1 mm and a height of 80 mm. A total of 180 complete cylindrical specimens, each measuring 39.1 mm in diameter and 80 mm in height, were prepared for this test. Specimens exceeding ±5 g in mass or ±1 mm in height were considered invalid and subsequently re-prepared, maintained, and inspected.

### 2.3. Test Equipment

**Figure 4:**
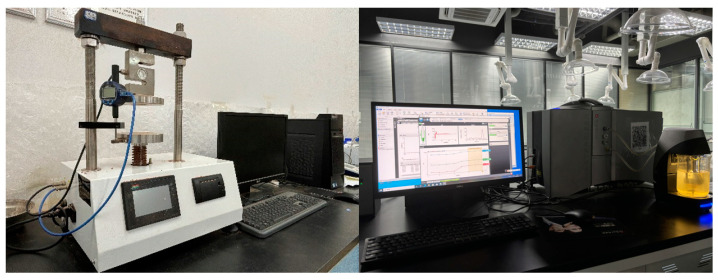
Unconfined Compressive Strength Test Equipment and Laser diffraction particle sizer.

The unconfined compressive strength test was conducted using a fully automatic unconfined compressive strength testing machine manufactured by TKA Limited (Nanjing, China), with a compression rate set at 1 mm/min, as shown in Figure 4. In accordance with the Standard for Geotechnical Test Methods (GB/T 50123-2019), the test was terminated when the axial force reached its peak or stabilized, with an axial strain of 3% to 5% applied to induce destructive strain. If a peak strength was observed, it was recorded as the ultimate compressive strength. In the absence of a peak, the strength corresponding to 20% of the axial strain was considered the ultimate compressive strength. To ensure data accuracy and minimize test errors, each test group included three specimens. Data with relatively large errors were excluded, and the average strength value of the remaining data was calculated to determine the final strength value.

The laser diffraction particle size analysis was conducted using a Malvern MAZ3000 (Westborough, MA, USA), which analyzed the particle size distribution (PSD) of the soil samples. The device operates over a range of 0.01 to 3500 μm. Following the measurements, the results were categorized into three groups based on the Unified Soil Classification System (USC) particle sizes: clay (<1.5 μm), silt (1.7–75 μm), and sand (>75 μm).

Scanning electron microscope (SEM) testing was conducted using a Hitachi Regulus 8100 model. Following sample preparation, a gold coating was applied to ensure sufficient electrical conductivity. The sample was then positioned on the test bench, and the lens height was adjusted to observe the images at various magnifications.

### 2.4. Experimental Programs

Unconfined compressive strength test is the ultimate stress of the specimen to resist axial pressure under the condition of no lateral pressure, and it is the main method to determine the value of natural strength of the soil and the sensitivity index of the soil. In this experiment, in order to study the strength characteristics and improve the strength index of recycled aggregate mixed soil material, we first configured the specimens of mixed red clay soil material with different maintenance age (7 d, 14 d, 28 d), different curing agent dosage (0%, 5%, 10%, 15%, 20%), and recycled aggregate dosage (0%, 20%, 40%, 60%), and at the same time, the maintenance age, the dosage of curing agent, and the recycled aggregate. The curing age, curing agent dosage, and recycled aggregate dosage were set to be labeled as M, C, and R, respectively, and the unconfined compressive strength test, scanning electron microscope (SEM) test, and laser diffraction particle size (PSD) test were carried out, and the results were analyzed mechanistically. The test program is now listed (see Table 3), in order to prevent test errors and ensure the reliability of the data, this experiment will be for each group of specimens to produce three independent replicates. Due to the curing agent dosage directly affecting the curing cost and curing effect, the curing agent dosage was set to 0%, 5%, 10%, 15%, and 20%. The recycled aggregate content, through the pre-experimentation of the approximate range of the experimental preparation of samples, was used in the preparation of samples for the optimal moisture content of red clay, the water dosage was set to the mass ratio, the curing agent dose is in the table, and the recycled aggregate was set to the mass ratio, namely, the ratio of the mass of the material used to the mass of the added red clay.

## 3. Results

To investigate the effects of the recycled aggregate, curing agent, and maintenance age on the unconfined compressive strength (*qu*), an experimental design scheme was implemented. This design allowed the determination of the unconfined compressive strength (*qu*) for each group of specimens. Due to the triplicate testing of each specimen, the average values of the unconfined compressive strength were analyzed to account for the various influencing factors.

### 3.1. Effect of Curing Agent on Unconfined Compressive Strength

**Figure 5:**
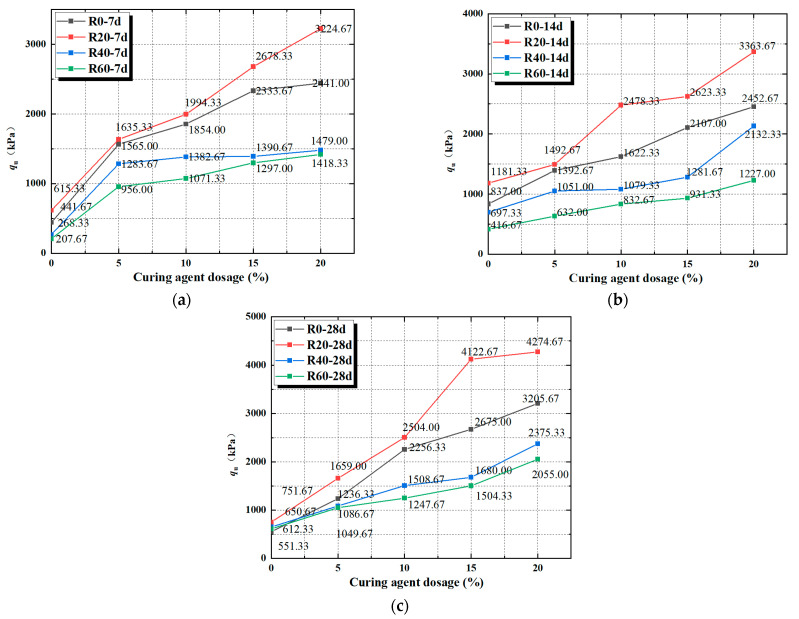
(**a**) The 7 d peak strain with different curing agent dosage; (**b**) 14 d peak strain with different curing agent dosage; (**c**) 28 d peak strain with different curing agent dosage.

One of the reasons for the change in unconfined compressive strength of mixed soil is due to the use of different dosages of soft soil curing agent in mixed soil; Figure 5a–c shows the effect of curing agent dosage on unconfined compressive strength. From Figure 5a–c, it can be seen that with the increase in the dosage of curing agent in the soil mix, the peak strength will show a gradual increase in the trend, and in the curing agent dosage of 20%, when the peak strength reaches the maximum. Similarly, it was found that the addition of a soft soil curing agent resulted in an increase in the unconfined compressive strength of the cured soil [40,41].

The peak strengths of C0R60-7d, C0R60-14d, and C0R60-28d specimens were minimized at 0% curing agent doping and were 207.67 kPa, 416.67 kPa, and 612.33 kPa, respectively. The peak strength increased with the increase in curing agent dosage, and at reaching 20%, the peak strengths of the C20R20-7d, C20R20-14d, and C20R20-28d specimens were all at their maximum values, which were 3224.67 kPa, 3363.67 kPa, and 4274.67 kPa, respectively. The strength values under the same conditions were increased by 424%, 184%, and 468%, respectively, compared to those at 0% curing agent doping. The peak strength increases with the increase in curing agent doping. The reason for this phenomenon is considered as follows: the active substances in the curing agent and the free water in the red clay produce a hydration reaction generated by the CSH, CAH, CASH, and other cementing substances; the cementing substances cross each other to form a spatial structure, and, at the same time, the soil particles in the red clay bond into a whole body that increases the strength of the curing specimen, and, at the same time, the main body, through the wrapping and bonding soil particles, strengthens the connection between soil particles, and changes the mechanical properties of soil while filling the pores of soil particles. The curing agent and red clay will consume a large amount of free water in the soil because the red clay used in this experiment has the optimal water content, so the curing agent dosage is from 0 to 20% of the process, and the specimen as a whole moves from a wet to dry hard state, which also leads to the curing agent dosage of the lower peak strength being relatively low, but with an increase in the dosage of the peak strength of the curing agent. Therefore, when the single factor is the curing agent dosage, a 20% curing agent dosage is the optimal dosage, which, at the same time, can ensure that the peak strength is the maximum value.

### 3.2. Effect of Recycled Aggregate on Unconfined Compressive Strength

**Figure 6:**
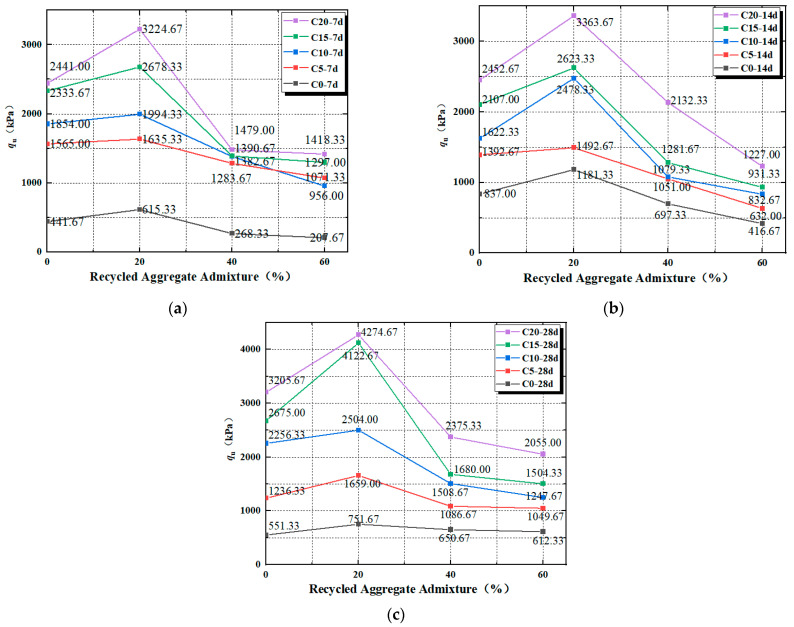
(**a**) The 7 d Peak strain with different recycled aggregate dosages; (**b**) 14 d Peak strain with different recycled aggregate dosages; (**c**) 28 d Peak strain with different recycled aggregate dosages.

The effect of the peak strength of mixed soil is closely related to the recycled aggregate dosing. Figure 6a–c shows the influence of the law of peak strength with the dosage of recycled aggregate. From Figure 6a–c, it can be seen that under the condition of the same maintenance age, with the increase in the recycled aggregate dosage, the unconfined compressive peak strength shows a trend of increasing and then decreasing. And under the condition of 7 d, 14 d, and 28 d of maintenance age, the peak strength reaches a maximum value of 3224.67 kPa, 3363.67 kPa, and 4274.67 kPa when the dosage of the recycled aggregate and curing agent reaches 20%; after that, with the addition of the dosage of recycled aggregate, the value of the peak strength decreases, and the minimum value of the peak strength is the peak strength of the recycled aggregate when 60% of the recycled aggregate is added. The minimum value is the peak strength corresponding to 60% of the recycled aggregate. Similar studies have demonstrated the impact of incorporating the recycled aggregate with a curing agent in soft soil [42,43].

The peak strength of the mixed soil is low when the recycled aggregate and curing agent are not added; as is shown in the figure, the peak strengths of the specimens numbered C0R0-7d, C0R0-14d, and C0R0-28d are 441.67kPa, 837 kPa, and 551.33 kPa, respectively; with the addition of recycled aggregate, the peak strength of the mixed soil reaches its maximum when the mixing amount of recycled aggregate reaches 20%, and the peak strength of the mixed soil reaches its maximum when the mixing amount of curing agent is zero, while the curing age is 7 d, 14 d, and 28 d. With the addition of the recycled aggregate, peak strength reaches its maximum when the dosage of the recycled aggregate in the soil mix reaches 20%, and when the dosage of the hardener reaches zero and the age of maintenance is 7 d, 14 d, and 28 d, the peak strengths of C0R20-7d, C0R20-14d, and C0R20-28d are 615.33 kPa, 1181.33 kPa, and 751.67 kPa, respectively; the peak strengths of the recycled aggregate decrease rapidly when the dosage of the recycled aggregate reaches 40–60%, the peak strengths of C0R60-7d, C0R60-14d, C0R60-28d, and C0R60-28d decrease rapidly, the C0R60-28d peak strengths at the same maintenance age were 207.57 kPa, 416.67 kPa, and 612.33 kPa, respectively.

The resulting peak strength of the soil mix must considered because when the regenerative aggregate doping is low, the soil particles in the soil mix will wrap the lesser doped regenerative aggregate to form agglomerates. Agglomerates in the specimen inside the formation of soil skeleton play a supportive role, and ultimately give the peak unconfined compressive strength; with the further increase in the dosage of regenerative aggregates, the specimen inside the red clay cannot be completely wrapped up with too many particles of regenerative aggregates, and at the same time, the regenerated aggregate, due to the close proximity between particles, form agglomerates between particles in mutual misalignment, so that the sample internal large particles produce misalignment, slip. On the other hand, due to the increase in the dosage of the recycled aggregate, the incomplete recycled aggregate wrapped around the soil particles will lead to the formation of more pores inside the specimen, resulting in a consequent decrease in the peak strength, and therefore the peak strength decreases rapidly when the recycled aggregate is 40% and 60%. At the same time, when mixed soil materials are added to the curing agent, soil particles fill the pore space, and, at the same time, the soft soil curing agent to varying degrees of cemented soil particles, as well as the regenerated aggregate, forms a support system. Relative to the specimen without the addition of the curing agent, curing agent dosing of 20% is seen when the unconfined peak compressive strength is relatively high; at the same time, for the specimen inside the regenerated aggregate of 20%, soil particles in the package of regenerated aggregate, and, at the same time, the curing agent produces a hydrate to fill smaller pore space, so that the specimen has a more crowded internal space. At the same time, when the specimen is 20% recycled aggregate, the soil particles are wrapped with recycled aggregate and the hydrates generated by the curing agent fill the smaller pores, which makes the specimen a more crowded internal space. From this, it can be analyzed that the peak unconfined compressive strength is at its maximum when the recycled aggregate dosage is 20% under the same conditions of maintenance age and curing agent dosage.

### 3.3. Effect of Age of Maintenance on Unconfined Compressive Strength

**Figure 7:**
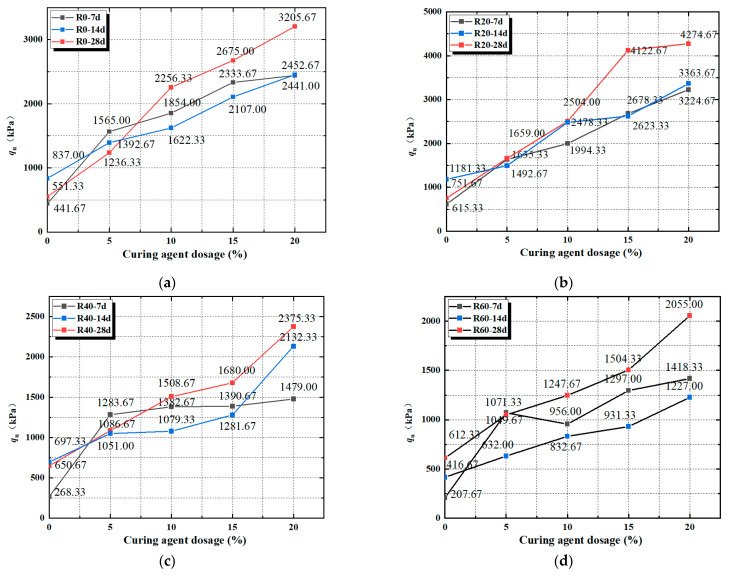
(**a**) Peak Strain at 0% Recycled Aggregate Admixture; (**b**) Peak Strain at 20% Recycled Aggregate Admixture; (**c**) Peak Strain at 40% Recycled Aggregate Admixture; (**d**) Peak Strain at 60% Recycled Aggregate Admixture.

The effect of maintenance age on peak strength is shown in Figure 7a–c, when the recycled aggregate dosage is consistent, the peak strength of the soil mix increases with the maintenance age and reaches a maximum with 28 d. It can also be seen that the recycled aggregate dosage reaches a maximum at 28 d of the maintenance age, whatever the value. Similar results have been observed regarding the effect of maintenance age on cement-cured soils [44].

Figure 7a–c show that the peak strength of the soil mix increases with the increase in hardener dosage and the peak strength of the soil mix specimen at 28 d is at its maximum as compared to the peak strength of the soil mix specimen at 7 d and 14 d. From the above analysis, it is clear that the peak strength of mixed soil remains at its maximum when the amount of curing agent is maintained at 20%, so when the amount of recycled aggregate is 0%, the 28 d peak strength is increased by 31.32% compared to 7 d peak strength; when the amount of recycled aggregate is 20%, the 28 d peak strength is increased by 32.56% compared to 7 d peak strength; when the amount of recycled aggregate is 40%, the 28 d peak strength is increased by 32.56% compared to 7 d peak strength; when the amount of recycled aggregate is 40%, the 28 d peak strength is increased by 32.56% compared to 7 d peak strength. The 28 d peak strength increased by 60.6% compared to the peak strength at 7 d; when the recycled aggregate dosage was 60%, the 28 d peak strength increased by 67.48% compared to the peak strength at 7 d.

The reason for this result is considered to be due to the increase in the amount of curing agent, curing agent hydration reaction will consume water inside the specimen, and with the age of maintenance from 7 d to 28 d, the process of the hydration reaction continues, so that the 7 d unconfined compressive strength peak is relatively small; when the hydration reaction continues to reach 14 d, the formation of cementing material in the soil mixture and soil particles form a dense agglomeration, and the unconfined compressive strength peak is relatively improved. The peak unconfined compressive strength is relatively elevated, and the hydration reaction inside the specimen is generally completed when the maintenance age is 28 d, and the specimen produces a more compact internal space structure, so the peak strength of the specimen reaches the maximum under compression at the maintenance age of 28 d. Therefore, 28 d is the most suitable age for the red clay under the optimal water content.

### 3.4. Mechanical Property Mechanism Analysis

**Figure 8:**
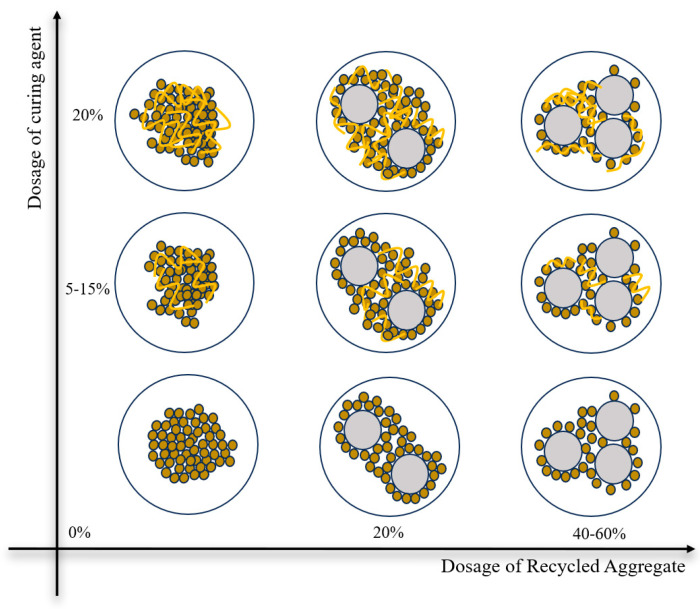
Schematic representation of changes in the particle skeleton.

Due to the mixture of red clay, recycled aggregate, and curing agent, the three will produce a joint action in the specimen to form a particle skeleton to bear the main compressive body part of this summary mainly through the analysis of no curing agent mixing, no recycled aggregate mixing, and recycled aggregate and curing agent at the same time under the action of the conditions of the specimen inside the particle skeleton to bear the main body of the change in the compressive body, as shown in Figure 8.

When there is no curing agent dosage, the peak strength of mixed soil will increase with the increase in recycled aggregate dosage, showing a trend of increasing and then decreasing. When the recycled aggregate is 20% it reaches its maximum; the reason for this phenomenon is that, with the increase in recycled aggregate dosage of mixed soil in the red clay wrapped in the recycled aggregate to form larger particles, the common assumption of the skeleton structure is to assume the unconfined compressive strength, because, due to the appropriate amount of recycled aggregate, the red clay can completely wrap the recycled aggregate to form relatively large particles, so the peak unconfined compressive strength is relatively large. However, when the amount of recycled aggregate is increased to 40~60%, the red clay particles in the soil mixture cannot completely wrap more recycled aggregate. When compressed by the red clay, the wrapped less recycled aggregate may produce contact between the particles to form a staggered, slippage, and, at the same time, due to the more particles of recycled aggregate, the red clay is not able to completely wrap the pore space generated within the sample, ultimately leading to the no-limit, where the peak compressive strength is relatively low.

When there is no regeneration aggregate doping, with the increase in curing agent doping in the mixed soil, its peak strength will show a rising trend all the time. When the doping of the curing agent is 5%, the hydration reaction that occurs in the curing agent is small, and the peak value of the unconfined compressive strength is also relatively small. With the increase in curing agent dosing up to 20%, the specimen inside the curing agent occurs in the hydration reaction of the cementing material, and together with the red clay, constitutes a skeleton structure. At this time, the compressive strength of the specimen is borne by the skeleton structure formed, and the peak unconfined compressive strength of the soil mixture reaches the maximum under the same conditions.

When the regenerated aggregate and curing agent dosage are 0%, the agglomerated red clay, as a mixture of soil internal particle skeleton structure, resists the specimen under pressure, so the peak strength is relatively low. With the increase in the regenerated aggregate and curing agent dosage, the internal specimen, at the same time, the regenerated aggregate and curing agent, when the regenerated aggregate dosage increased to 20%, curing agent dosage to 20%, the randomly distributed regenerated aggregate within the specimen. With the red clay and curing agent fully wrapped, red clay more fully agglomerated to the outside recycled aggregate gradually wrapped into larger skeleton particles, while the skeleton particles by the curing agent reaction produced by the cementing material to fill its pores make the specimen more crowded, so that the specimen can withstand a higher pressure. At this stage, the skeleton structure is assumed by the red clay, the cementing material, and the recycled aggregate encapsulated by the red clay, which ultimately maximizes the peak strength. As the recycled aggregate increases again to 40–60%, the red clay and the cementing material produced by the curing agent cannot fill the pore structure produced by the excess recycled aggregate in contact with each other inside the specimen, thus resulting in more pore space inside the specimen, and, at the same time, the recycled aggregates are in contact with each other, which produces a certain amount of misalignment when the specimen is compressed and ultimately leads to a relatively low peak strength of the specimen.

### 3.5. Particle Size Distribution

**Figure 9:**
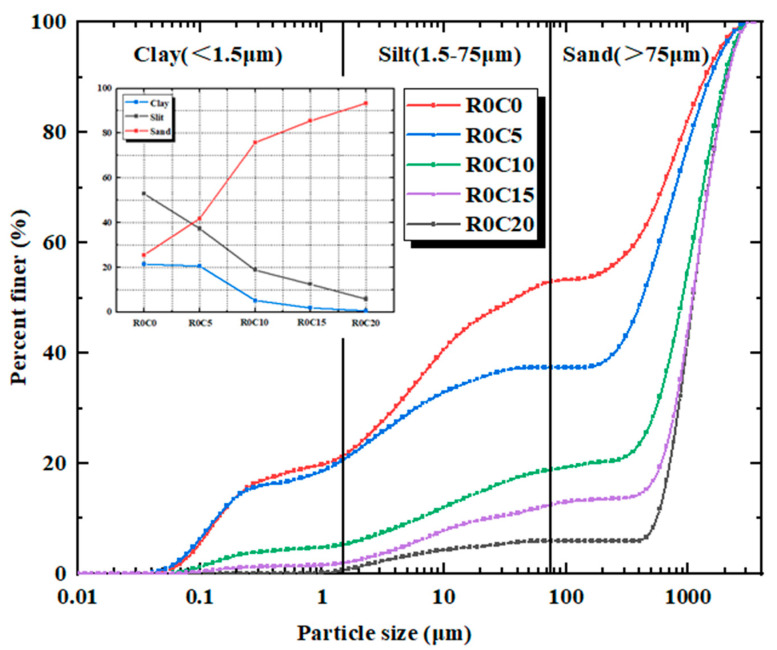
Particle size distribution curves for different curing agent dosages with 0% recycled aggregate.

Particle size distribution (PSD) is a fundamental property and classification of soils, which has an important impact on various engineering properties. In conjunction with this experimental program, a laser particle size analyzer was used to measure the change in particle size of different curing agents with red clay under no addition of recycled aggregate. Figure 9 shows the particle size distribution curves of different curing agent dosages under 0% dosage of recycled aggregate and the trend of the percentage of pink clay.

As shown in Figure 9, the results indicate that when the PSD curve in the red clay is shifted to the right with the increase in the amount of curing agent doping, which indicates that the content of coarse particles as a percentage of the mixed soil increases with the increase in the amount of curing agent doping. In the figure, it can be clearly seen that with the increase in curing agent dosage, the proportion of coarse particles also increased significantly, and the PSD curve of the mixed soil was divided into two different phases with 10% curing agent dosage as the turning point, and in this process, the agglomeration effect of the mixed soil continued to increase, and showed an increasing trend of coarse particles and the decrease in fine particles. The clay and powder particles of the R0C5 specimen were reduced by 3.5% and 29.4%, respectively, and the corresponding sand particles increased by 63.5%. It has been shown that large particles of agglomerates are produced within the specimen after the curing agent is mixed in the soil [34]. When the curing agent dosage reached 20%, the specimen viscous and powder particles decreased by 65.6% and 52.8%, respectively, and the corresponding sand particles increased by 9.2%. When the curing agent dosage reaches 20%, it is obvious that the percentage of coarse aggregate is greater. The reason for this phenomenon is that as the amount of hardener in the soil mix increases, the reaction between the hardener and the red clay produces a cementing substance that bonds with the red clay particles to form agglomerates, resulting in a relatively large proportion of coarse particles. Overall, the addition of the curing agent to the soil mix resulted in an increase in the number of large soil aggregates and hence an increase in the peak unconfined compressive strength.

### 3.6. Field Emission Scanning Electron Microscope (FESEM)

**Figure 10:**
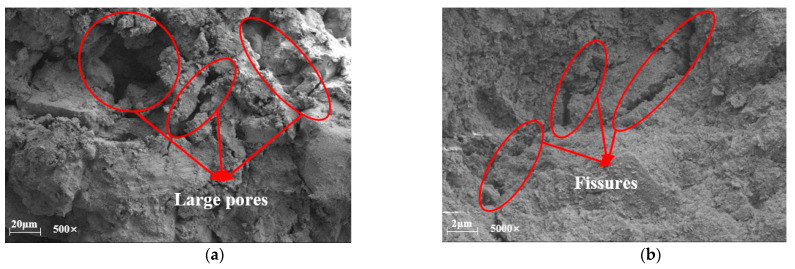
R0C0-SEM micrographs (**a**,**b**).

**Figure 11:**
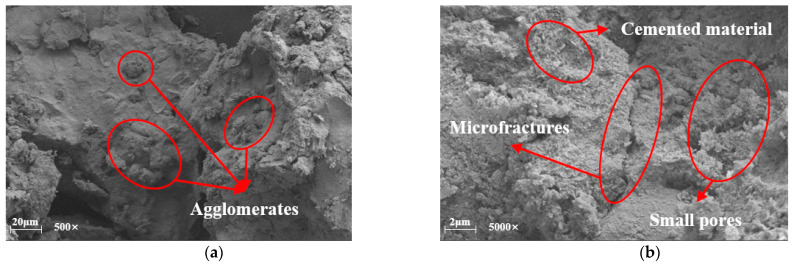
R0C10-SEM micrographs (**a**,**b**).

**Figure 12:**
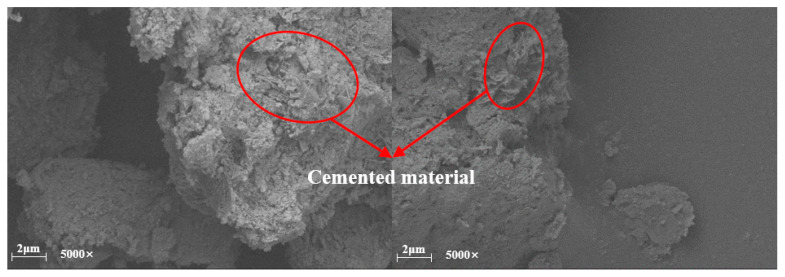
Micrograph of R0C20-SEM.

The enhancement mechanism of the peak unconfined compressive strength of mixed soils under the action of curing agents was revealed by field emission scanning electron microscopy. Since the recycled aggregate particles are huge particles compared to the red clay, this summary analyzes the mechanism of action by observing the SEM micrographs of the specimens (R0C0-28d, R0C10-28d, R0C20-28d) without the addition of recycled aggregate. Through Figure 10, Figure 11 and Figure 12, it can be found that with the increase in the amount of curing agent, the cementing material in the soil mixture increases, and the macroscopic manifestation is the increase in the peak value of the unconfined compressive strength.

Figure 10a,b show specimen R0C0-28d respectively magnified 500 times and 5000 times after the microstructure; in Figure 10a, it can be seen that between the soil particles are many pore structures, and the pores are relatively small, in the micrograph of the small particles between the effect of agglomeration, and small particles formed by the formation of agglomeration have relatively more contact points. Figure 10b shows that the specimen produced more cracks within the specimen. Figure 10b shows that there are more cracks inside the specimen and the cracks are longer, so the peak strength reached by the specimen under pressure is relatively small. Figure 11a,b are the microstructure diagrams of the test specimen R0C10-28d after magnification of 500 times and 5000 times, respectively. In Figure 11a, a more compact structural form is shown, and the effect of agglomeration between the soil particles is more obvious, and, in Figure 11b, it can be seen that due to the addition of the curing agent, the cementing material and the clay particles form a more compact agglomeration, with the pore structure greatly reduced. At the same time, the inter-particle cementing effect effectively fills the pore space inside the test specimen, so the peak strength achieved by the specimen under pressure is relatively high. Figure 12 shows the microstructure of specimen R0C20-28d after magnification by 5000 times, in which it is obvious that there is the presence of cemented material, and the interaction between the soil particles and the cemented material makes the specimen peak unconfined compressive strength reach the maximum.

## 4. Conclusions

The aim of this study was to investigate the mechanical properties of mixed soils with the simultaneous addition of curing agent and recycled aggregate at a specific age of maintenance. Red clay with specific water content was used to prepare standard unconfined compressive strength specimens by mixing with different dosages of curing agent (0%, 5%, 10%, 15%, 20%), and recycled aggregate (0%, 20%, 40%, 60%), and unconfined compressive strength tests, PSD tests, and scanning electron microscope (SEM) tests were carried out on all of the samples, and the following conclusions were drawn:

(a). The PSD curve of the mixed soil was gradually shifted to the right by the addition of curing agent to the red clay, and the shifting trend was more obvious with the increase in curing agent dosage. When the amount of curing agent reaches 20%, the proportion of coarse grains reaches the maximum, which indicates that the addition of curing agent not only changes the particle size in the mixed soil, but also induces the aggregation effect of red clay particles. This phenomenon proves that the curing agent can enhance the mechanical properties of the mixed soil from a microscopic aspect.
(b). Using SEM scanning electron microscope (Thermo Fisher Scientific, Waltham, MA, USA) to scan and analyze the micrographs of specimens without added recycled aggregates (R0C0-28d, R0C10-28d, R0C20-28d), it was concluded that the cementing material produced by the hydration reaction of the curing agent inside the specimens and the red clay worked together to fill the pore structure and cracks of the specimens to make the specimens more compact, which further proved the mechanical properties of the curing agent-enhanced soil mixes from the microscopic point of view. The mechanical properties of the curing agent-enhanced mixed soil were proved.
(c). Due to the addition of the recycled aggregate and curing agent, the mechanical properties of the soil mixture in the two dosages of 20% reached the maximum, which was considered because of the random distribution of the recycled aggregate inside the specimen by the red clay and the curing agent fully wrapped agglomerates, while the curing agent reaction of the cementing material was produced by the filling of its pores, so that the specimen was more crowded inside the specimen, so that the specimen can withstand higher pressures, and ultimately lead to the peak strength reaching the maximum.
(d). Through the unconfined compressive strength test, and through the one-factor peak analysis method, the following conclusions were found: the maximum peak strength when the dosage of recycled aggregate was 20%, the maximum peak strength when the dosage of hardener was 20%, and the maximum peak strength when the maintenance time was 28 d. Therefore, for this study, the proportion program R20C20-28d was the optimal program, which provides a reference value for the actual project.

## Figures and Tables

**Table 1:** Basic physical properties of red clay.

Relative Density of Soil/(g·cm^−3^)	Liquid Limit/%	Plastic Limit/%	Plasticity Index	Optimum Moisture Content/%
2.71	50.1	22.7	27.4	20.1

**Table 2:** Chemical composition of soft soil curing agent (unit: %).

Material Type	Calcite	Tricalcium Silicate	Calcium Phosphorite	Dicalcium Silicate
%	9.5	44	5.9	40.6

**Table 3:** Design solutions for unconfined compressive testing.

Moisture Content	Recycled Aggregate Admixture (R)	Dosage of Soft Soil Curing Agent (C)	Age of Conservation (M)
20%	0%	0%, 5%, 10%, 15%, 20%	7 d, 14 d, 28 d
20%	20%		7 d, 14 d, 28 d
20%	40%		7 d, 14 d, 28 d
20%	60%		7 d, 14 d, 28 d

## Data Availability

The original contributions presented in the study are included in the article, further inquiries can be directed to the corresponding author.
